# A solitary wave solution to the generalized Burgers-Fisher's equation using an improved differential transform method: A hybrid scheme approach

**DOI:** 10.1016/j.heliyon.2021.e07001

**Published:** 2021-05-12

**Authors:** Timilehin Kingsley Akinfe, Adedapo Chris Loyinmi

**Affiliations:** Department of Mathematics, Tai Solarin University of Education, Ijagun, Ijebu ode, Ogun state, Nigeria

**Keywords:** Solitary wave solutions, Hybrid scheme, Asymptotic methods, Elzaki projected differential transform, Burgers-Fisher's equation, Reaction-mechanisms, Reaction-diffusion equations, 3D plots

## Abstract

In this research, an unrivalled hybrid scheme which involves the coupling of the new Elzaki integral transform (an improved version of Laplace transform) and a modified differential transform called the projected differential transform (PDTM) have been implemented to solve the generalized Burgers-Fisher's equation; which springs up due to the fusion of the Burgers' and the Fisher's equation; describing convective effects, diffusion transport or interaction between reaction mechanisms, traffic flows; and turbulence; consequently finding meaningful applicability in the applied sciences viz: gas dynamics, fluid dynamics, turbulence theory, reaction-diffusion theory, shock-wave formation, traffic flows, financial mathematics, and so on.

Using the proposed Elzaki projected differential transform method (EPDTM), a generalized exact solution (Solitary solution) in form of a Taylor multivariate series has been obtained; of which the highly nonlinear terms and derivatives handled by PDTM have been decomposed without expansion, computation of Adomian or He's polynomials, discretization, restriction of parameters, and with less computational work whilst achieving a highly convergent results when compared to other existing analytical/exact methods in the literature, via comparison tables, 3D plots, convergence plots and fluid-like plots. Thus showing the distinction, novelty and huge advantage of the proposed method as an asymptotic alternative, in providing generalized or solitary wave solution to a wider class of differential equations.

## 1 Introduction

One of the militating problems faced in the field of computational mathematics, numerical analysis, and applied sciences is the problem of obtaining an exact solution to models, be it linear or nonlinear. This makes it easier to study and understand the dynamical behaviour or pattern a model exhibits. Apparently, every model developed or formulated needs to be solved so as to obtain a realistic relationship between the variables or parameters of the model (It could be independent or dependent), to investigate or study the slight change in the model when any of these parameters or variables in question is varied (either increased or decreased), and to also presume the long term effect of the model system. Virtually all of these models have been built on differential equations of which a larger proportion of these models are built on Nonlinear differential equations (Ordinary or Partial differential equation of the nonlinear type).

Wikipedia the online encyclopedia defined a differential equation as a mathematical equation that relates some function with its derivatives [1]. In applications, the functions usually represent physical quantities, the derivatives represent their rates of change, and the equation defines a relationship between the two. An ordinary differential equation (ODE) is an equation which consists of functions of single variables with their total derivatives, while a partial differential equation (PDE) is that which consists of several independent variables and a dependent variable with partial derivatives. For this reason, when we are investigating a phenomenon with a single variable and that is time dependent; for example population dynamics over a period of time, the oscillation of a pendulum bob for a specific period of time, and so on; the ordinary differential equation (Linear or nonlinear) is put to play in formulating a suitable model for these phenomena. On the other hand, when more variables including time are involved in a phenomenon, for example the flow of a fluid in a channel is determined by several variables like the temperature of the fluid, viscosity, pressure, the nature of the channel, and so on; then the partial differential equation comes to play here.

As a result of the complexity of nature, virtually all processes and phenomenon in sciences and engineering are inherently nonlinear of which they are being described by nonlinear partial differential equation as there are several conditions and parameters to be considered in the system. This is why partial differential equations most especially the nonlinear partial differential equation has craved the attention numerous mathematicians and researchers in the applied sciences. The nonlinear partial differential equations are often used to describe variety of processes and real life phenomenon viz: genetic configuration, mutation and variation as in Fisher's equation, Magnetic flux, intensity, and quantum field theory as in sine Gordon equation, the waves and patterns produced by shallow waters as in Korteweg-De-Vries (KdV) equation, advection-diffusion mechanisms and dynamics as in the Burgers-Huxley's equation, and so on.

These nonlinear partial differential equations are known to be difficult when it comes to obtaining their exact solutions. Thus, more attention and care is paid to these equations in constructing and implementing an appropriate technique, scheme, method or algorithm in solving them. This is to say that exact solution rarely exists for nonlinear partial differential equations.

Numerous researchers have studied the nonlinear ordinary and partial differential equations over the years [2], [3], [4], [5], [6], [7], [8], [9], [10], but consistent findings still yield highly pertinent results and recommendations, as there is no best method or algorithm in providing exact solutions to an equation. Nonlinear PDEs can be classified as the integrable and non-integrable [6], [7] depending the nature of the equation in question.

The integrable equations are those having their behaviour determined by their initial conditions and they can be simply solved by integrating them from those conditions. In some cases, some non-integrable nonlinear PDEs are integrable after some symbolic transformation. Some of them are: the sine-Gordon equations applicable in magnetic flux, intensity, and quantum field theory, and solitons; the Schrödinger equation applicable in optics and water waves, the dispersionless equations, and so on.

On the other hand, the non-integrable equations namely: the Burger-Huxley equation applicable in advection-diffusion models, the Ginsburg-Landau equation which is prominent in the theory of conductivity, the Yang-Mills equation, the Burgers-Fisher's equation and so on, have few or no exact solutions as they require special treatment in constructing valid, reliable and efficient methods, scheme or algorithms in order to solve them.

The investigation and development of travelling wave solutions and solitary wave's theory have played a prominent role in the nonlinear science in understanding the asymptotic patterns of solutions and dynamical behaviours of developed models by seeking a solitary wave solution to them. This in fact has made the world of computational mathematics flourish excellently.

In 1834, John Scott Russell was the first to observe the solitary waves when he watched a large protrusion of water slowly travelling on the Edinburgh-Glasgow canal without change in shape. Since then, diverse scientific views have been obtained over time from numerous researchers and mathematicians [11], [12], [13], [14]. Consequently, it is interesting to point out that there is no precise definition of a soliton. However, a soliton can be defined as a solution of a nonlinear partial differential equation that exhibits the following properties viz: the solution should demonstrate a wave of permanent form; the solution is localized, which means that it either decays exponentially to zero such as the solutions provided by the KdV equation, or converges to a constant at infinity such as the solitons given by the sine-Gordon equation; the soliton interacts with other solitons preserving its character [15], [16], [17].

Solitary solutions are basically problem-specific, implying that some particular methods or schemes are much suitable for some particular equations or models. Solitary wave solution is implementing an algorithm, scheme, method, or a technique on some highly nonlinear partial differential equations with its variables and parameters; in order to obtain a generalized solution of the problem. This makes it easy to examine and study the generalized behaviour of such model by varying the equation parameters.

Since the nonlinear partial differential equation (most especially the non-integrable) requires special attention and methods in solving them. Then solitary solution becomes effective and comes to play here. Several remarkable methods viz: Hirota Bilinear method, the inverse scattering method, tanh-function method, Weierstrass elliptic method, exponential-function method, the first integral method, and so on [11], have been used to obtain solitary solutions to numerous nonlinear models.

Apart from these standard analytical methods mentioned, several asymptotic methods have been used by mathematicians to obtain solitary wave solutions of some highly nonlinear equations viz: Adomian decomposition method by Abdul-Majid Wazwaz [18], variational iteration method by M. Javidi and A. Golbabai [19], Laplace decomposition method by Arun Kumar and Ram Dayal Pankaj [20] and so on.

Several methods have been established in the literature over the years for solving nonlinear partial differential equations including the Burgers-Fisher's equation viz: the Taylor collocation method [21], [22], wavelet collocation method [23], [24], cubic B-spline method [25], [26], [27], differential quadrature method [28], homotopy analysis method (HAM) [29], [30], [31], reduced differential transform method (RDTM) [32], [33], new iterative method (NIM) [34], [35], Sumudu decomposition method (SDM) [36], Elzaki decomposition method (EDM) [37], [38], perturbation iteration method (PIM) [39], variational iteration method (VIM) [40], [41], Adomian decomposition method (ADM) [18], [42], [43], Elzaki homotopy transformation perturbation method (EHTPM) [6], [7], homotopy perturbation transformation method (HPTM) [44], Elzaki differential transform [45], Elzaki projected differential transform method (EPDTM) [46], new iterative transform method [47], Laplace Adomian decomposition method (LADM) [48], Laplace variational iteration method [49], homotopy analysis transform method [50], and so on.

All these methods have their respective radius of convergence which implies that the validity, reliability, and efficacy of a method when implemented any of these models or equations differ. In this case, it is worth it to convey a convergence analysis on any scheme, method, algorithm or technique being implemented on a problem so as to reveal its authenticity. Loyinmi A.C. & Akinfe T.K. (2019) [6] buttressed this and conveyed some convergence and error analysis on an algorithm being implemented in their work.

The subsequent organization of this work is structured as follows: Section 2 provides a detailed description of the Burgers-Fisher's equation and its applicability in numerous fields, section 3 and 4 gives explicit details and description of the two methods (Elzaki transform and the projected differential transform method), section 5 illustrates the application of the hybrid method Elzaki homotopy transformation perturbation method (EHTPM) on the generalized form of the B-F equation with δ≠0,1, section 6 establishes the implementation of the Elzaki projected differential transform method (EPDTM) in providing a solitary solution for the Burgers-Fisher's equation and its generalized form, while the results with variation in the model parameters were presented via tables and plots in sections 7 and 8, lastly, the discussion of findings and conclusion were elucidated in sections 9 and 10 respectively.

## 2 The Burgers-Fisher's equation (B-F equation)

The Burgers-Fisher's equation (B-F equation) emanated from the fusion of the well-known equations of Martinus Burger and Harry Bateman (the Burgers' equation); and Ronald Fisher (Fisher's equation).

The Burgers-Fisher's equation and its generalized form are encountered and it is of high importance for describing different mechanisms in applied sciences. The Burgers-Fisher's equation arises in financial mathematics, gas dynamics, traffic flow, applied mathematics and physical applications. The equation is a prototypical model for describing the interaction between reaction mechanisms, convection effects, and diffusion transports.

The generalized form of Burgers-Fisher's equation is a prominent equation in the field of nonlinear dynamics and appears in numerous applications such as shock waves formation, fluid dynamics, heat conduction, turbulence, traffic flow, gas dynamics, sound wave in viscous medium and some other applications in the field of applied sciences.

The generalized B-F equation is of the form:

(1) $$\frac{\partial u}{\partial t}+\alpha u^{\delta }\frac{\partial u}{\partial x}-\frac{\partial^{2}u}{\partial x^{2}}=\beta u\left(1-u^{\delta }\right)$$

or

(2) $$u_{t}+\alpha u^{\delta }u_{x}=\beta u\left(1-u^{\delta }\right)$$

∀x∈(0,1),t≥0.

Subject to the initial condition

(3) $$u\left(x,0\right)={\left\{\frac{1}{2}+\frac{1}{2}\tanh\left[\frac{-\alpha \delta }{2\left(\delta +1\right)}x\right]\right\}}^{\frac{1}{\delta }}$$

Earlier, Hassan N.A. Ismail et al. (2004) [51] provided an exact solution to the equations (1) and (2) using the Adomian decomposition method as:

(4) $$u(x,t)={\left\{\frac{1}{2}+\frac{1}{2}\tanh\left[\frac{-\alpha \delta }{2\left(\delta +1\right)}\left(x-\left(\frac{\alpha }{\delta +1}+\frac{\beta \left(\delta +1\right)}{\alpha }\right)t\right)\right]\right\}}^{\frac{1}{\delta }}$$

From the equations (1) and (2), it will interest you to know that:

When δ=1, we obtain the main B-F equation for any selected value of *α* and *β*;

When δ=1 and β=0, the generalized Burgers equation is obtained;

When δ=1 and α=0, we obtain the Huxley's equation;

When δ=1, α=0, and β=1 the Fisher's equation is obtained;

When δ=1, α=0, and β=0, we obtain the one dimensional heat conduction equation.

All these parameter variations giving rise to distinct equations with distinct properties which makes the Burgers-Fisher's equation an interesting equation to be looked into.

Most recently, Loyinmi Adedapo C. and Akinfe Timilehin K. (2020) implemented an algorithm using the Elzaki transform to provide exact solutions to the Burgers-Huxley equation of three distinct cases as a result of variation in the equation parameters [7]. Again in (2019), using a hybrid algorithm involving Elzaki transform and homotopy perturbation method (EHTPM), they proffered exact solution to the family of Fisher's reaction-diffusion equation which is well applicable in genetics, stochastic processes, nuclear reactor theory, and so on. See Ref. [6].

Several researches have been carried out in proffering an exact solution to nonlinear partial differential equations including the Burger-Fisher's equation [9], [10], [51], [52], [53]. Majority of these solutions obtained to nonlinear partial differential equations are usually in multivariate series form converging rapidly to the exact solution of the problem.

In this research work, we have implemented an unprecedented hybrid method viz: ‘the projected differential transform’ coupled with the modification of the Laplace and Sumudu transform called the ‘Elzaki transform’ on the generalized Burger-Fisher's equation of the form

(5) $$\frac{\partial u}{\partial t}+\alpha u\frac{\partial u}{\partial x}-\frac{\partial^{2}u}{\partial x^{2}}=\beta u\left(1-u\right)$$

Subjected to the initial condition:

(6) $$u\left(x,0\right)=\frac{e^{\mu x}}{e^{\mu x}+e^{-\mu x}}$$

with $\mu =-\frac{\alpha }{4}$;

The equation (5) here gives the same result as when δ=1,α=α,β=β in equation (1) and the results obtained using our proposed method are in form an infinite multivariate series which converges to close form that is a replica of the exact solution of the problem.

## 3 The Elzaki integral transform

Elzaki transform is a new integral transform was invented by Tarig M. Elzaki [54] and was derived from the classical Fourier integral. It is a modified version of the Laplace and existing Sumudu transform. Based on the mathematical simplicity of the Elzaki transform, it facilitates the process of solving ordinary and partial differential equations in the time domain [54], [55], [56], [57].

As a result, Elzaki integral transform is a powerful and efficient tool that has provided analytical/exact solution to some differential equations which Sumudu transform has failed to solve. See [58].

Due to this efficiency and the fact that Elzaki transform is the modification of Laplace and Sumudu transform, we have preferred to use the Elzaki transform to other integral transforms coupled with the projected differential transform method as this is method unprecedented.

The Elzaki transform is a semi-infinite convergent integral of the form.

(7) $$T\left(v\right)=v\int_{0}^{\infty }f(t)e^{-\frac{t}{v}}dt$$

or

(8) $$T\left(v\right)=v^{2}\int_{0}^{\infty }f(vt)e^{-t}dt$$

The function *f* is of exponential order in the set

(9) $$A=\{f(t):∋m,k_{1},k_{2},>0,|f(t)|<Me^{\frac{\left|t\right|}{k_{j}}}$$

If

$$t\in {\left(-1\right)}^{j}X[0,\infty ]\}$$

then

$$E\left\{f(t)\right\}=T\left(v\right)=v\int_{0}^{\infty }f(t)e^{-\frac{t}{v}}dt$$

It is called modified Sumudu transform invented/introduced by Tarig M. Elzaki.

By proceeding, we have the transform of derivatives using integration by parts:

(10) $$E\left[\frac{\partial f}{\partial t}\right]=\frac{1}{v}T\left(x,v\right)-vf\left(v,0\right)$$

(11) $$E\left[\frac{\partial^{2}f}{\partial t^{2}}\right]=\frac{1}{v^{2}}T\left(x,v\right)-f\left(x,0\right)-v\frac{\partial f\left(x,0\right)}{\partial t}$$

(12) $$E\left[\frac{\partial f}{\partial x}\right]=T^{\prime }\left(x,v\right)=\frac{dT\left(X,0\right)}{dx}$$

(13) $$E\left[\frac{\partial f}{\partial x}\right]=T^{\prime \prime }\left(x,v\right)=\frac{d^{2}T\left(X,0\right)}{dx^{2}}$$

Higher order derivatives with respect to *t* can be obtained by mathematical induction as

(14) $$E\left[\frac{\partial^{n}f(x.t)}{\partial t^{n}}\right]\Rightarrow \frac{E\left[f(x,t)\right]}{v^{n}}-\sum_{k=0}^{n-1}v^{2-n+k}\frac{\partial^{k}f(x,0)}{\partial t^{k}}$$

## 4 The projected differential transform (PDTM): an improved differential transform (DTM)

The projected differential transform method (PDTM) is the modified and improved version of the well known differential transform method (DTM) developed by Bongsoo Jang (2010) [59] to proffer exact solution to linear and nonlinear initial value problems (IVPs). The DTM invented by Zhou (1986), Wuhan, China, [60] of which he used to solve problems in Electrical circuits, is close to the Taylor series, but it's different from the conventional high-order Taylor series in determining coefficients. The DTM has been employed to solve many important problems in science and engineering fields and obtain accurate approximations or asymptotic solutions [59]. However, it also has some difficulties due to the nonlinearity in some highly nonlinear problems but not in the case of PDTM.

PDTM is an effective asymptotic method which is valid and reliable in handling nonlinear terms in differential equations. It decomposes the nonlinear terms and derivatives in a differential manner such that there is ‘zero need’ for computing, expanding, and comparing coefficients for some special polynomials like in the case of He's polynomial in the Homotopy perturbation method (HPM) and Adomian polynomials in Adomian decomposition method (ADM).

PDTM has no discretization, restriction of parameters, and assumption of parameters, yet yielding highly convergent results with less computational work when compared with other asymptotic or semi-analytic methods in the literature. The decomposed nonlinear terms obtained from the PDTM are the same as that obtained when computing polynomials and comparing coefficients for computed polynomials in Adomian decomposition method (ADM) and Homotopy perturbation method (HPM) respectively. PDTM has been used effectively in previous literature by numerous researchers and mathematicians who have found it more valid, reliable, and convergent on wider classes of nonlinear partial differential equations [61], [62], [63].

Definition 4.1 The projected differential transform U(X,k) of u(X,t) with respect to the variable *t* at $t_{0}$ is defined by:

(15) $$U\left(X,k\right)=\frac{1}{k!}{\left[\frac{\partial^{k}}{\partial t^{k}}u\left(X,t\right)\right]}_{t=t_{0}}$$

$$X=\left(x_{1},x_{2},x_{3},\ldots ,x_{n}\right)$$

where u(X,t) is the unknown function from the problem and U(X,k) is the transformed function of u(X,t).

While the inverse transform of u(X,k) with respect to the variable *t* at $t_{0}$ is defined by:

(16) $$u\left(X,t\right)=\sum_{k=0}^{\infty }U\left(X,k\right){\left(t-t_{0}\right)}^{k}$$

Now by combining equations (15) and (16), we have

(17) $$u\left(X,t\right)=\sum_{k=0}^{\infty }\frac{1}{k!}{\left[\frac{\partial^{k}}{\partial t^{k}}u\left(X,t\right)\right]}_{t=t_{0}}{\left(t-t_{0}\right)}^{k}$$

From the above definitions, the fundamental operations of the PDTM are given by the following theorem.

Theorem 4.2 *Let* P(X,k)*,* Q(X,k)*, and* R(X,k) *be the projected differential transforms of the functions* p(X,t)*,* q(X,t)*, and* r(X,t) *respectively, with* $X=\left(x_{1},x_{2},x_{3},\ldots ,x_{n}\right)$*, then*

1. ***Linearity Property of PDTM***
  *If* r(X,t)=αp(X,t)+βq(X,t)*,*
  *then* R(X,k)=αP(X,k)+βQ(X,k)
  *where α and β*
2. ***PDTM of Products***
  *If* r(X,t)=p(X,t)⋅q(X,t)*,*
  *then* $R\left(X,k\right)=\sum_{\varphi_{1}=0}^{k}P\left(X,\varphi_{1}\right)Q\left(X,k-\varphi_{1}\right)$
3. ***PDTM of Multiple Products***
  *Suppose we have three or more functions to be transformed such that:*
  $$r\left(X,t\right)=p_{1}\left(X,t\right)\cdot p_{2}\left(X,t\right)\cdot p_{3}\left(X,t\right)\cdot p_{4}\left(X,t\right)\ldots \cdot p_{n}\left(X,t\right),$$
  *then*
  $$\begin{array}{c} R\left(X,k\right)=\sum_{k_{n-1}=0}^{k}\sum_{k_{n-2}=0}^{k_{n-1}}\sum_{k_{n-3}=0}^{k_{n-2}}\cdots \sum_{k_{2}=0}^{k_{3}}\sum_{k_{1}=0}^{k_{2}}P_{1}\left(X,k_{1}\right)P_{2}\left(X,k_{2}-k_{1}\right)\\ \times \ldots \times P_{n-2}\left(X,k_{n-2}-k_{n-3}\right)P_{n-1}\left(X,k_{n-1}-k_{n-2}\right)P_{n}\left(X,k-k_{n-1}\right) \end{array}$$
4. ***PDTM of time derivatives***
  *If* $r\left(X,t\right)=\frac{\partial^{n}}{\partial t^{n}}p\left(X,t\right)$*,*
  *then*
  R(X,k)=(k+1)(k+2)…(k+n)P(X,k+n)
  $$=\frac{\left(k+n\right)!}{k!}P\left(X,k+n\right),\;n\in \left\{1,2,3,\ldots \right\}.$$
5. ***PDTM of space derivative***
  *If* $r\left(X,t\right)=\frac{\partial^{n}}{\partial x_{i}^{n}}p\left(x_{1},x_{2},x_{3},...,x_{n},t\right)$*,*
  *then*
  $$R\left(X,k\right)=\frac{\partial^{n}}{\partial x_{i}^{n}}P\left(x_{1},x_{2},x_{3},...,x_{n},k\right),i\in \left\{1,2,...,n\right\},\;n\in \left\{1,2,...\right\}.$$
6. ***PDTM for the product of variables and time with indices***
  *If* $r\left(X,t\right)=x_{1}^{\alpha_{1}}x_{2}^{\alpha_{2}}x_{3}^{\alpha_{3}}\ldots x_{n}^{\alpha_{n}}t^{\alpha_{m}}$*,*
  *then*
  $$R\left(X,k\right)=x_{1}^{\alpha_{1}}x_{2}^{\alpha_{2}}x_{3}^{\alpha_{3}}\ldots x_{n}^{\alpha_{n}}\delta \left(k_{m}-\alpha_{m}\right)=\;\left\{ \begin{array}{c} x_{1}^{\alpha_{1}}x_{2}^{\alpha_{2}}x_{3}^{\alpha_{3}}\ldots x_{n}^{\alpha_{n}},\;k_{m}=\alpha_{m}\\ 0,\;\text{otherwise} \end{array}\right.$$
7. ***PDTM for the product of variables, problem function, and time***
  *If* $r\left(X,t\right)=x_{1}^{\alpha_{1}}x_{2}^{\alpha_{2}}x_{3}^{\alpha_{3}}\ldots x_{n}^{\alpha_{n}}t^{\alpha_{m}}u\left(X,t\right)$*,*
  *then*
  $$R\left(X,k\right)=x_{1}^{\alpha_{1}}x_{2}^{\alpha_{2}}x_{3}^{\alpha_{3}}\ldots x_{n}^{\alpha_{n}}U\left(X,k-n\right).$$

## 5 Implementation of the proposed scheme (EPDTM) on the generalized Burgers-Fisher's equation

Consider the Burgers-Fisher's equation in the equation (1) given as:

(18) $$\frac{\partial u}{\partial t}+\alpha u^{\delta }\frac{\partial u}{\partial x}-\frac{\partial^{2}u}{\partial x^{2}}=\beta u\left(1-u^{\delta }\right)$$

Subject to the initial condition

(19) $$u\left(x,0\right)={\left\{\frac{1}{2}+\frac{1}{2}\tanh\left(\frac{-\alpha \delta }{2\left(\delta +1\right)}x\right)\right\}}^{\frac{1}{\delta }}$$

The equation (18) can be re-arranged as:

(20) $$\frac{\partial u}{\partial t}=\frac{\partial^{2}u}{\partial x^{2}}-\alpha u^{\delta }\frac{\partial u}{\partial x}+\beta u\left(1-u^{\delta }\right)$$

By applying the Elzaki transform to the equation (20) we have

(21) $$E\left[\frac{\partial u}{\partial t}\right]=E\left[\frac{\partial^{2}u}{\partial x^{2}}-\alpha u^{\delta }\frac{\partial u}{\partial x}+\beta u\left(1-u^{\delta }\right)\right]$$

From the Elzaki of derivatives, we have that

(22) $$E\left[\frac{\partial u}{\partial t}\right]=\frac{E\left[u\left(x,t\right)\right]}{v}-vu\left(x,0\right)$$

Then the equation (21) becomes

(23) $$E\left[u\left(x,t\right)\right]=v^{2}u\left(x,0\right)+vE\left[\frac{\partial^{2}u}{\partial x^{2}}-\alpha u^{\delta }\frac{\partial u}{\partial x}+\beta u\left(1-u^{\delta }\right)\right]$$

By taking the inverse Elzaki of (23) we have

(24) $$u\left(x,t\right)=u\left(x,0\right)+E^{-1}\left[vE\left[\frac{\partial^{2}u}{\partial x^{2}}-\alpha u^{\delta }\frac{\partial u}{\partial x}+\beta u\left(1-u^{\delta }\right)\right]\right]$$

(25) $$u\left(x,0\right)={\left\{\frac{1}{2}+\frac{1}{2}\tanh\left(\frac{-\alpha \delta }{2\left(\delta +1\right)}x\right)\right\}}^{\frac{1}{\delta }}$$

But this initial condition above appears to be a hyperbolic function, and from the theory of hyperbolic functions; let $-\frac{\alpha \delta }{2\left(\delta +1\right)}=\mu$; we obtain the transformation and simplification of equation (25) as:

(26) $$u\left(x,0\right)={\left[\frac{e^{\mu x}}{e^{\mu x}+e^{-\mu x}}\right]}^{\frac{1}{\delta }}$$

Inserting this initial condition into equation (24) we have

(27) $$u\left(x,t\right)={\left[\frac{e^{\mu x}}{e^{\mu x}+e^{-\mu x}}\right]}^{\frac{1}{\delta }}+E^{-1}\left[vE\left[\frac{\partial^{2}u}{\partial x^{2}}-\alpha u^{\delta }\frac{\partial u}{\partial x}+\beta u\left(1-u^{\delta }\right)\right]\right]$$

We now implement the next scheme which is the projected differential transform on the equation (27) as:

(28) $$u\left(x,k+1\right)={\left[\frac{e^{\mu x}}{e^{\mu x}+e^{-\mu x}}\right]}^{\frac{1}{\delta }}+E^{-1}\left[vE^{-1}\left[\begin{array}{c} \frac{\partial^{2}u\left(x,k\right)}{\partial x^{2}}-\alpha {\left(u\left(x,k\right)\right)}^{\delta }\frac{\partial u\left(x,k\right)}{\partial x}+\beta u\left(x,k\right)\\ -\beta {\left(u\left(x,k\right)\right)}^{\delta +1} \end{array}\right]\right]$$

The above equation (28) can be miniaturized as:

(29) $$u\left(x,k+1\right)={\left[\frac{e^{\mu x}}{e^{\mu x}+e^{-\mu x}}\right]}^{\frac{1}{\delta }}+E^{-1}\left[vE^{-1}\left[P_{k}-Q_{k}+R_{k}-S_{k}\right]\right]$$

where $P_{k}=P\left(x,k\right),Q_{k}=Q\left(x,k\right),R_{k}=R\left(x,k\right),S_{k}=S\left(x,k\right)$ are the projected differential transforms of nonlinear terms $\frac{\partial^{2}u}{\partial x^{2}}$, $\alpha u^{\delta }\frac{\partial u}{\partial x}$, *βu*, and $\beta u^{\delta +1}$.

From the equation (29), we have

(30) $$\begin{array}{c} u\left(x,0\right)={\left[\frac{e^{\mu x}}{e^{\mu x}+e^{-\mu x}}\right]}^{\frac{1}{\delta }}\\ u\left(x,1\right)=E^{-1}\left[vE^{-1}\left[P_{0}-Q_{0}+R_{0}-S_{0}\right]\right],\\ u\left(x,2\right)=E^{-1}\left[vE^{-1}\left[P_{1}-Q_{1}+R_{1}-S_{1}\right]\right],\\ u\left(x,3\right)=E^{-1}\left[vE^{-1}\left[P_{2}-Q_{2}+R_{2}-S_{2}\right]\right],\\ \vdots \\ u\left(x,n\right)==E^{-1}\left[vE^{-1}\left[P_{n-1}-Q_{n-1}+R_{n-1}-S_{n-1}\right]\right] \end{array}$$

Then, the solution of the equation (20) is given as:

(31) $$u\left(x,t\right)=\sum_{k=0}^{\infty }u(x,k)$$

To illustrate the capability and simplicity of this scheme, we now implement this method on the generalized Burgers-Fisher's equation in (20) with δ=1 as a solitary solution example.

## 6 Solitary solution to the Burgers-Fisher's equation using the Elzaki projected differential transform method (illustration)

Consider the generalized Burgers-Fisher's equation in (18) with δ=1 as:

(32) $$\frac{\partial u}{\partial t}+\alpha u\frac{\partial u}{\partial x}-\frac{\partial^{2}u}{\partial x^{2}}=\beta u\left(1-u\right)$$

The equation (32) is then subjected to the initial condition

(33) $$u\left(x,0\right)=\frac{e^{-\frac{\alpha }{4}x}}{e^{\frac{\alpha }{4}x}+e^{-\frac{\alpha }{4}x}}$$

Let $\mu =-\frac{\alpha }{4}$; then we can also write the initial condition as:

(34) $$u\left(x,0\right)=\frac{e^{\mu x}}{e^{\mu x}+e^{-\mu x}}$$

Here, we would be using either of these representations above interchangeably for easy computation in the quest of solving this problem

By taking the Elzaki of equation (32) we have

(35) $$E\left[\frac{\partial u}{\partial t}\right]=E\left[\frac{\partial^{2}u}{\partial x^{2}}-\alpha u\frac{\partial u}{\partial x}+\beta u\left(1-u\right)\right]$$

(36) $$∴E\left[u\left(x,t\right)\right]=v^{2}u\left(x,0\right)+vE\left[\frac{\partial^{2}u}{\partial x^{2}}-\alpha u\frac{\partial u}{\partial x}+\beta u\left(1-u\right)\right]$$

By taking the inverse Elzaki transform of (36), we have

(37) $$u\left(x,t\right)=u\left(x,0\right)+E^{-1}\left[vE\left[\frac{\partial^{2}u}{\partial x^{2}}-\alpha u\frac{\partial u}{\partial x}+\beta u\left(1-u\right)\right]\right]$$

(38) $$\Rightarrow u\left(x,t\right)=\frac{e^{-\frac{\alpha }{4}x}}{e^{\frac{\alpha }{4}x}+e^{-\frac{\alpha }{4}x}}+E^{-1}\left[vE\left[\frac{\partial^{2}u}{\partial x^{2}}-\alpha u\frac{\partial u}{\partial x}+\beta u-\beta u^{2}\right]\right]$$

By applying the next scheme which is the projected differential transform on eq. (38) we obtain

(39) $$u\left(x,k+1\right)=\frac{e^{-\frac{\alpha }{4}x}}{e^{\frac{\alpha }{4}x}+e^{-\frac{\alpha }{4}x}}+E^{-1}\left[vE^{-1}\left[\begin{array}{c} \frac{\partial^{2}u\left(x,k\right)}{\partial x^{2}}-\alpha \sum_{r=0}^{k}u\left(x,r\right)\frac{\partial u\left(x,k-r\right)}{\partial x}+\beta u\left(x,k\right)\\ -\beta \sum_{r=0}^{k}u\left(x,r\right)u\left(x,k-r\right) \end{array}\right]\right]$$

In order to miniaturize the equation (39) above, let the transformed nonlinear terms $\frac{\partial^{2}u\left(x,k\right)}{\partial x^{2}};\;\alpha \sum_{r=0}^{k}u\left(x,r\right)\frac{\partial u\left(x,k-r\right)}{\partial x};\;\beta u\left(x,k\right)$; and

$\beta \sum_{r=0}^{k}u\left(x,r\right)u\left(x,k-r\right)$ be denoted by $P_{k}=P\left(x,k\right)$, $Q_{k}=Q\left(x,k\right)$, $R_{k}=R\left(x,k\right)$, $S_{k}=S\left(x,k\right)$ respectively:

(40) $$∴u\left(x,k+1\right)=\frac{e^{-\frac{\alpha }{4}x}}{e^{\frac{\alpha }{4}x}+e^{-\frac{\alpha }{4}x}}+E^{-1}\left[vE^{-1}\left[P_{k}-Q_{k}+R_{k}-S_{k}\right]\right]$$

From the equation (40) we obtain

(41) $$u\left(x,0\right)=\frac{e^{-\frac{\alpha }{4}x}}{e^{\frac{\alpha }{4}x}+e^{-\frac{\alpha }{4}x}}$$

(42) $$u\left(x,1\right)=E^{-1}\left[vE^{-1}\left[P_{0}-Q_{0}+R_{0}-S_{0}\right]\right]$$

By computing all PDTM terms in equation (42) we have

$$P_{0}=\frac{\partial^{2}u\left(x,0\right)}{\partial x^{2}};Q_{0}=\alpha u\left(x,0\right)\frac{\partial u\left(x,0\right)}{\partial x};R_{0}=\beta u\left(x,0\right);S_{0}=\beta {\left(u\left(x,0\right)\right)}^{2}$$

(43) $$∴u\left(x,1\right)=E^{-1}\left[vE^{-1}\left[\frac{\partial^{2}u\left(x,0\right)}{\partial x^{2}}-\alpha u\left(x,0\right)\frac{\partial u\left(x,0\right)}{\partial x}+\beta u\left(x,0\right)-\beta {\left(u\left(x,0\right)\right)}^{2}\right]\right]$$

By evaluating and simplifying the decomposed derivative and nonlinear terms in the equation (43) accordingly, we obtain

(44) $$P_{0}=-{\left(\frac{\alpha }{4}\right)}^{2}\frac{4\left(e^{\frac{\alpha }{4}x}-e^{-\frac{\alpha }{4}x}\right)}{{\left(e^{\frac{\alpha }{4}x}+e^{-\frac{\alpha }{4}x}\right)}^{3}};Q_{0}=\frac{\frac{\alpha }{2}e^{\mu x}}{{\left(e^{\frac{\alpha }{4}x}+e^{-\frac{\alpha }{4}x}\right)}^{3}};R_{0}=\frac{\beta e^{-\frac{\alpha }{2}x}}{e^{\frac{\alpha }{4}x}+e^{-\frac{\alpha }{4}x}};S_{0}=\frac{\beta e^{-\frac{\alpha }{2}x}}{{\left(e^{\frac{\alpha }{4}x}+e^{-\frac{\alpha }{4}x}\right)}^{2}}$$

Now let $\eta_{0}=P_{0}-Q_{0}+R_{0}-S_{0}$

(45) $$∴\eta_{0}=-{\left(\frac{\alpha }{4}\right)}^{2}\frac{4\left(e^{\frac{\alpha }{4}x}-e^{-\frac{\alpha }{4}x}\right)}{{\left(e^{\frac{\alpha }{4}x}+e^{-\frac{\alpha }{4}x}\right)}^{3}}-\frac{2\mu e^{\mu x}}{{\left(e^{\frac{\alpha }{4}x}+e^{-\frac{\alpha }{4}x}\right)}^{3}}+\frac{\beta e^{\mu x}}{e^{\frac{\alpha }{4}x}+e^{-\frac{\alpha }{4}x}}-\frac{\beta e^{2\mu x}}{{\left(e^{\frac{\alpha }{4}x}+e^{-\frac{\alpha }{4}x}\right)}^{2}}$$

By simplifying (45) accordingly and completely, we have

(46) $$\eta_{0}=-\frac{\left(e^{\frac{\alpha }{4}x}+e^{-\frac{\alpha }{4}x}\right)\beta -\frac{\alpha^{2}}{2}e^{\frac{\alpha }{4}x}+\frac{\alpha^{2}}{4}\left(e^{-\frac{\alpha }{4}x}-e^{\frac{\alpha }{4}x}\right)}{{\left(e^{-\frac{\alpha }{4}x}-e^{\frac{\alpha }{4}x}\right)}^{3}}$$

Put the simplified equations (46) in (43) we obtain

(47) $$u\left(x,1\right)=E^{-1}\left[vE^{-1}\left[-\left[\frac{\left(e^{\frac{\alpha }{4}x}+e^{-\frac{\alpha }{4}x}\right)\beta -\frac{\alpha^{2}}{2}e^{\frac{\alpha }{4}x}+\frac{\alpha^{2}}{4}\left(e^{-\frac{\alpha }{4}x}-e^{\frac{\alpha }{4}x}\right)}{{\left(e^{-\frac{\alpha }{4}x}-e^{\frac{\alpha }{4}x}\right)}^{3}}\right]\right]\right]$$

By computing the terms and evaluating the Elzaki operators in the equation (47) accordingly, we have the solution term $u_{1}\left(x,t\right)=u\left(x,1\right)$ as;

(48) $$u\left(x,1\right)=-\frac{\left(e^{\frac{\alpha }{4}x}+e^{-\frac{\alpha }{4}x}\right)\beta -\frac{\alpha^{2}}{2}e^{\frac{\alpha }{4}x}+\frac{\alpha^{2}}{4}\left(e^{-\frac{\alpha }{4}x}-e^{\frac{\alpha }{4}x}\right)}{{\left(e^{-\frac{\alpha }{4}x}-e^{\frac{\alpha }{4}x}\right)}^{3}}t$$

Next, we obtain the next term u(x,2) in the series solution

For k=1 in the equation (40), we have

(49) $$u\left(x,2\right)=E^{-1}\left[vE^{-1}\left[P_{1}-Q_{1}+R_{1}-S_{1}\right]\right]$$

Similarly, by evaluating the decomposed terms $P_{1}$, $Q_{1}$, $R_{1}$, and $S_{1}$ accordingly as the previous computation we have

$$P_{1}=P\left(x,1\right)=\frac{\partial^{2}u\left(x,1\right)}{\partial x^{2}}=-\left(\frac{\alpha^{2}}{4}\right)\frac{1}{{\left(e^{\mu x}+e^{-\mu x}\right)}^{5}}\left[\begin{array}{c} \left[\frac{\alpha^{2}}{4}+\beta \right]\left(e^{\frac{3\alpha }{4}x}+e^{-\frac{3\alpha }{4}x}\right)+\left[3\beta -2\alpha^{2}\right]e^{-\frac{\alpha }{4}x}\\ +\left[3\beta +\frac{9\alpha^{2}}{4}\right]e^{\frac{\alpha }{4}x} \end{array}\right]t$$

(50) $$∴P_{1}=-\left(\frac{\alpha^{2}}{4}\right)\frac{1}{{\left(e^{\mu x}+e^{-\mu x}\right)}^{5}}\left[\begin{array}{c} \left[\frac{\alpha^{2}}{4}+\beta \right]\left(e^{\frac{3\alpha }{4}x}+e^{-\frac{3\alpha }{4}x}\right)+\left[3\beta -2\alpha^{2}\right]e^{-\frac{\alpha }{4}x}\\ +\left[3\beta +\frac{9\alpha^{2}}{4}\right]e^{\frac{\alpha }{4}x} \end{array}\right]t$$

(51) $$Q_{1}=\alpha \left(\sum_{r=0}^{k=0}u\left(x,r\right)\frac{\partial u\left(x,k-r\right)}{\partial x}+\sum_{r=1}^{k=1}u\left(x,r\right)\frac{\partial u\left(x,k-r\right)}{\partial x}\right)$$

(52) $$\Rightarrow Q_{1}=\frac{2\alpha \beta \left[\frac{\alpha^{2}-2\alpha -4\beta }{4}\right]e^{-\frac{3\alpha }{4}x}+2\alpha \beta \left[\frac{\alpha^{2}+4\beta }{4}\right]e^{-\frac{\alpha }{4}x}+\alpha \beta \left[\alpha^{2}+4\beta \right]e^{\frac{\alpha }{4}x}}{{\left(e^{\frac{\alpha }{4}x}+e^{-\frac{\alpha }{4}x}\right)}^{5}}t$$

(53) $$R_{1}=\beta u\left(x,1\right)$$

(54) $$∴R_{1}=\frac{\left[\left(\frac{\alpha^{2}\beta }{4}+\beta^{2}\right)\left(e^{\frac{\alpha }{4}x}+e^{-\frac{\alpha }{4}x}\right)\right]}{{\left(e^{\frac{\alpha }{4}x}+e^{-\frac{\alpha }{4}x}\right)}^{4}}t$$

(55) $$S_{1}=\beta \left(u\left(x,0\right)u\left(x,1\right)+u\left(x,1\right)u\left(x,0\right)\right)=2\beta u_{0}u_{1}$$

(56) $$∴S_{1}=-\frac{\left[2\alpha^{2}\beta^{2}+4\beta^{2}\alpha +2\beta^{2}\right]e^{-\frac{\alpha }{2}x}-2\beta \left(\beta +\alpha^{2}\right)}{{\left(e^{\frac{\alpha }{4}x}+e^{-\frac{\alpha }{4}x}\right)}^{4}}t$$

Let

(57) $$\begin{array}{c} \eta_{1}(x)=-\left(\frac{\alpha^{2}}{4}\right)\frac{1}{{\left(e^{\mu x}+e^{-\mu x}\right)}^{5}}\left[\begin{array}{c} \left[\frac{\alpha^{2}}{4}+\beta \right]\left(e^{\frac{3\alpha }{4}x}+e^{-\frac{3\alpha }{4}x}\right)+\left[3\beta -2\alpha^{2}\right]e^{-\frac{\alpha }{4}x}\\ +\left[3\beta +\frac{9\alpha^{2}}{4}\right]e^{\frac{\alpha }{4}x} \end{array}\right]-\\ \frac{2\alpha \beta \left[\frac{\alpha^{2}-2\alpha -4\beta }{4}\right]e^{-\frac{3\alpha }{4}x}+2\alpha \beta \left[\frac{\alpha^{2}+4\beta }{4}\right]e^{-\frac{\alpha }{4}x}+\alpha \beta \left[\alpha^{2}+4\beta \right]e^{\frac{\alpha }{4}x}}{{\left(e^{\frac{\alpha }{4}x}+e^{-\frac{\alpha }{4}x}\right)}^{5}}\\ +\frac{\left[\left(\frac{\alpha^{2}\beta }{4}+\beta^{2}\right)\left(e^{\frac{\alpha }{4}x}+e^{-\frac{\alpha }{4}x}\right)\right]}{{\left(e^{\frac{\alpha }{4}x}+e^{-\frac{\alpha }{4}x}\right)}^{4}}+\frac{\left[2\alpha^{2}\beta^{2}+4\beta^{2}\alpha +2\beta^{2}\right]e^{-\frac{\alpha }{2}x}-2\beta \left(\beta +\alpha^{2}\right)}{{\left(e^{\frac{\alpha }{4}x}+e^{-\frac{\alpha }{4}x}\right)}^{4}} \end{array}$$

From equation (40), when k=1 we obtain

(58) $$u\left(x,2\right)=E^{-1}\left[vE^{-1}\left[P_{1}-Q_{1}+R_{1}-S_{1}\right]\right]$$

The equation (58) can be written as:

(59) $$u\left(x,2\right)=E^{-1}\left[vE^{-1}\left[\eta_{1}(x)t\right]\right]$$

By simplifying the terms in $\eta_{1}(x)$ accordingly and evaluating the Elzaki operators in the equation (59) above we have

(60) $$u_{2}\left(x,t\right)=-\frac{1}{2}\frac{1}{{\left(e^{\frac{\alpha }{4}x}+e^{-\frac{\alpha }{4}x}\right)}^{5}}\left[\begin{array}{c} \left(\beta^{2}-\frac{3\alpha^{2}}{16}-\frac{\alpha^{2}\beta }{2}\right)e^{-\frac{3\alpha }{4}}-\left(\frac{\alpha^{4}}{16}+\frac{\alpha^{2}\beta }{2}+\beta^{2}\right)e^{\frac{3\alpha }{4}}\\ -\left(\frac{19\alpha^{4}}{16}+\frac{\alpha^{2}\beta }{2}-\beta^{2}\right)e^{-\frac{3\alpha }{4}x}+\left(\frac{7\alpha^{4}}{16}+\frac{3\alpha^{2}\beta }{2}-\beta^{2}\right)e^{\frac{\alpha }{4}x} \end{array}\right]t^{2}$$

Similarly, we obtain other solution terms $u_{3}\left(x,t\right),u_{4}\left(x,t\right)$ and so on.

Thus, we have a generalized solution of the Burgers-Fisher's equation in equations (18) and (20) as:

(61) $$\begin{array}{c} U\left(x,t\right)=\frac{e^{-\frac{\alpha }{4}x}}{e^{\frac{\alpha }{4}x}+e^{-\frac{\alpha }{4}x}}-\frac{\left(e^{\frac{\alpha }{4}x}+e^{-\frac{\alpha }{4}x}\right)\beta -\frac{\alpha^{2}}{2}e^{\frac{\alpha }{4}x}+\frac{\alpha^{2}}{4}\left(e^{-\frac{\alpha }{4}x}-e^{\frac{\alpha }{4}x}\right)}{{\left(e^{-\frac{\alpha }{4}x}-e^{\frac{\alpha }{4}x}\right)}^{3}}t\\ -\frac{1}{2}\frac{1}{{\left(e^{\frac{\alpha }{4}x}+e^{-\frac{\alpha }{4}x}\right)}^{5}}\left[\begin{array}{c} \left(\beta^{2}-\frac{3\alpha^{2}}{16}-\frac{\alpha^{2}\beta }{2}\right)e^{-\frac{3\alpha }{4}}-\left(\frac{\alpha^{4}}{16}+\frac{\alpha^{2}\beta }{2}+\beta^{2}\right)e^{\frac{3\alpha }{4}}\\ -\left(\frac{19\alpha^{4}}{16}+\frac{\alpha^{2}\beta }{2}-\beta^{2}\right)e^{-\frac{3\alpha }{4}x}+\left(\frac{7\alpha^{4}}{16}+\frac{3\alpha^{2}\beta }{2}-\beta^{2}\right)e^{\frac{\alpha }{4}x} \end{array}\right]t^{2}+\cdots \end{array}$$

This gives the solitary, exact or generalized solution of the Burgers-Fisher's equation which corresponds to the solution obtained using the normal analytical and asymptotic means in the literature.

The validity, reliability of this solution; and the authenticity of this method implemented have been buttressed in the convergence plots in this research.

## 7 Results

Here, we validate the authenticity and efficacy of the proposed Elzaki Projected Differential transform method (EPDTM) in providing a solitary or generalized solution to the Burgers-Fisher's equation (B-F equation) by comparing results of the exact solution and that of our proposed EPDTM.

The exact results here were that obtained by authors of prominent literatures viz: Hassan N.A. Ismail et al. (2004) and M. Mestrovic et al. (2017) [51], [64], [65] using Adomian decomposition method, and Jiang Lu using the First integral method. Furthermore, we have also compared the current proposed method (EPDTM) with the Reduced differential transform method (RDTM) results obtained in D. Kocacoban et al. (2011) [33] in Table 3 for α=β=0.001 and γ=1 as stated previously.

This exact solution of the generalized Burgers-Fisher's equation in equation (1) is given by:

$$u(x,t)={\left\{\frac{1}{2}+\frac{1}{2}\tanh\left[\frac{-\alpha \delta }{2\left(\delta +1\right)}\left(x-\left(\frac{\alpha }{\delta +1}+\frac{\beta \left(\delta +1\right)}{\alpha }\right)t\right)\right]\right\}}^{\frac{1}{\delta }}$$

For δ=1, we obtain

(62) $$u(x,t)=\left\{\frac{1}{2}+\frac{1}{2}\tanh\left[-\frac{\alpha }{4}\left(x-\left(\frac{\alpha }{2}+\frac{2\beta }{\alpha }\right)t\right)\right]\right\}$$

### 7.1 Table 1

We present the exact and asymptotic or approximate results of the Burgers-Fisher's equation in equations (61) and (62) respectively when α=−1 and β=2 at x=1,x=2,x=3 for each value of t=0.1,0.2,0.3,0.4,0.5 which is similar to the asymptotic computation in [66] using the homotopy perturbation method.

**Table 1:** The table of exact results, present hybrid scheme results (EPDTM), and absolute error of the scheme when *β* = 2, *α* = −1, for *x* = 1,2,3 and *t* = 0.1 to 0.5.

*x*	*t*	Exact	EPDTM	Error = |Exact-EPDTM|
x=1	0.1	0.67370709	0.67370709	0.00000000
	0.2	0.72111517	0.72111517	0.00000000
	0.3	0.76404759	0.76404758	0.00000001
	0.4	0.80218388	0.80218358	0.0000003
	0.5	0.83548353	0.83547986	0.00000037
x=2	0.1	0.77294225	0.77294226	0.00000001
	0.2	0.80999843	0.80999843	0.00000000
	0.3	0.84224131	0.84224130	0.00000001
	0.4	0.86989152	0.86989128	0.00000024
	0.5	0.89330940	0.89330684	0.00000256
x=3	0.1	0.84877174	0.84877174	0.00000000
	0.2	0.87544664	0.87544664	0.00000000
	0.3	0.89798193	0.89798193	0.00000000
	0.4	0.91682730	0.91682743	0.00000013
	0.5	0.93245330	0.93245482	0.00000152

### 7.2 Table 2

We present the exact and asymptotic or approximate results of the Burgers-Fisher's equation in equations (61) and (62) respectively when α=3 and β=6 at x=1,x=2,x=3 for each value of t=0.1,0.2,0.3,0.4,0.5.

**Table 2:** The table of exact results, present hybrid scheme results (EPDTM), and absolute error of the scheme when *β* = 6, *α* = 3, for *x* = 1,2,3 and *t* = 0.1 to 0.5.

*x*	*t*	Exact	EPDTM	Error = |Exact-EPDTM|
x=1	0.1	0.33737816	0.33737819	0.00000003
	0.2	0.53742984	0.53742989	0.00000005
	0.3	0.72611498	0.72611425	0.00000073
	0.4	0.85814893	0.85814896	0.00000003
	0.5	0.93245330	0.93245317	0.00000013
x=2	0.1	0.10201806	0.10201806	0.00000000
	0.2	0.20587037	0.20587017	0.00000002
	0.3	0.37168381	0.37168337	0.00000044
	0.4	0.57444251	0.57444670	0.00000419
	0.5	0.75491498	0.75491486	0.00000012
x=3	0.1	0.02472269	0.02472269	0.00000000
	0.2	0.05468131	0.05468158	0.00000027
	0.3	0.11660296	0.11660265	0.00000031
	0.4	0.23147521	0.23147521	0.00000000
	0.5	0.13296424	0.13296435	0.00000011

### 7.3 Table 3

In order to buttress our scheme, we present the exact and asymptotic or approximate results (EPDTM and RDTM) of the Burgers-Fisher's equation in equations (61) and (62) respectively when α=0.001 and β=0.001 at x=0.01,x=0.04,x=0.08 for each value of t=0.02,0.04,0.06,0.08.

**Table 3:** The table of exact results, present hybrid scheme results (EPDTM), a similar asymptotic technique (RDTM), absolute error of the present scheme and the absolute error of RDTM when *β* = 0.001, *α* = 0.001, for *x* = 0.01,0.04,0.08 and *t* = 0.02,0.04,0.06,0.08.

*x*	*t*	Exact	EPDTM	RDTM	EPDTM Error = |Exact-EPDTM|	RDTM Error = |Exact-RDTM|
x=0.01	0.02	0.5000000006	0.5000000375	0.5000500000	3.69 × 10^−8^	5.0 × 10^−5^
	0.04	0.4999975006	0.5000008752	0.5000007500	3.4 × 10^−6^	3.2 × 10^−6^
	0.06	0.4999950006	0.5000001375	0.5000100000	5.1 × 10^−7^	1.5 × 10^−5^
	0.08	0.4999925006	0.5000001875	0.5000012500	7.7 × 10^−6^	7.7 × 10^−6^
x=0.04	0.02	0.5000075025	0.5000001200	0.500012500	7.4 × 10^−6^	5.0 × 10^−6^
	0.04	0.5000050025	0.5000050025	0.500015000	0	9.9 × 10^−6^
	0.06	0.5000025025	0.5000100038	0.500017500	7.5 × 10^−6^	1.7 × 10^−6^
	0.08	0.5000000025	0.5000150050	0.500020000	1.5 × 10^−5^	2.0 × 10^−5^
x=0.08	0.02	0.5000175050	0.5000175050	0.500022500	0	5.0 × 10^−6^
	0.04	0.5000150050	0.5000000025	0.500025000	1.5 × 10^−5^	9.9 × 10^−5^
	0.06	0.5000125050	0.5000050038	0.500027500	7.5 × 10^−6^	1.5 × 10^−5^
	0.08	0.5000100050	0.5000100050	0.500030000	0	2.0 × 10^−5^

### 7.4 Table 4

We present the exact and asymptotic or approximate results of the Burgers-Fisher's equation in equations (61) and (62) respectively when α=0.5 and β=0.01 at x=1,x=2,x=3 for each value of t=0.1,0.2,0.3,0.4,0.5.

**Table 4:** The table of exact results, present hybrid scheme results (EPDTM), and absolute error of the scheme when *β* = 0.01, *α* = 0.5, for *x* = 1,2,3 and *t* = 0.1 to 0.5.

*x*	*t*	Exact	EPDTM	Error = |Exact-EPDTM|
x=1	0.1	0.4396087682	0.4396087680	0.0000000002
	0.2	0.4413956012	0.4413956011	0.0000000001
	0.3	0.4431839532	0.4431839530	0.0000000002
	0.4	0.4449737792	0.4449737791	0.0000000001
	0.5	0.4467650338	0.4467650337	0.0000000001
x=2	0.1	0.3792459522	0.3792459521	0.0000000001
	0.2	0.3809542242	0.3809542235	0.0000000007
	0.3	0.3826654473	0.3826654429	0.0000000044
	0.4	0.3843795844	0.3843795841	0.0000000003
	0.5	0.3860965975	0.3860965969	0.0000000006
x=3	0.1	0.3224030874	0.3224030870	0.0000000004
	0.2	0.3239889526	0.3329889518	0.0000000008
	0.3	0.3255788703	0.3255788701	0.0000000002
	0.4	0.3271728142	0.3271728126	0.0000000016
	0.5	0.3287707574	0.3287707524	0.0000000050

## 8 Convergence, three dimensional (3D), and fluid-like plots

**Figure 1:**
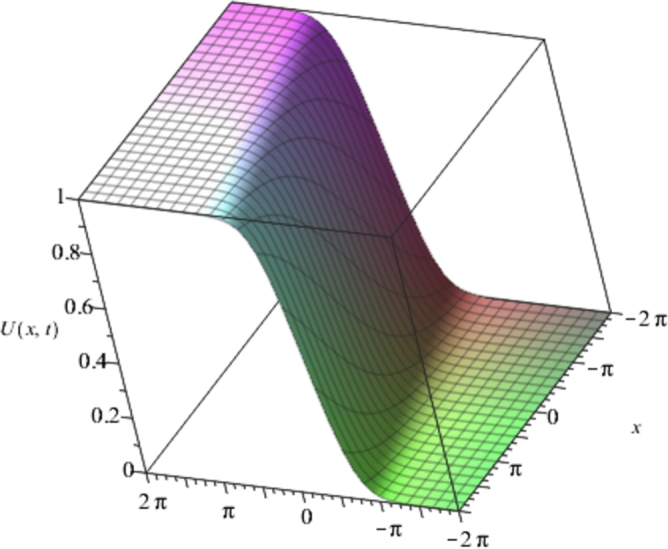
Solution plots of the exact solution/closed form solution in eq. (62) with ***α* = −1**, ***β* = 2**.

**Figure 2:**
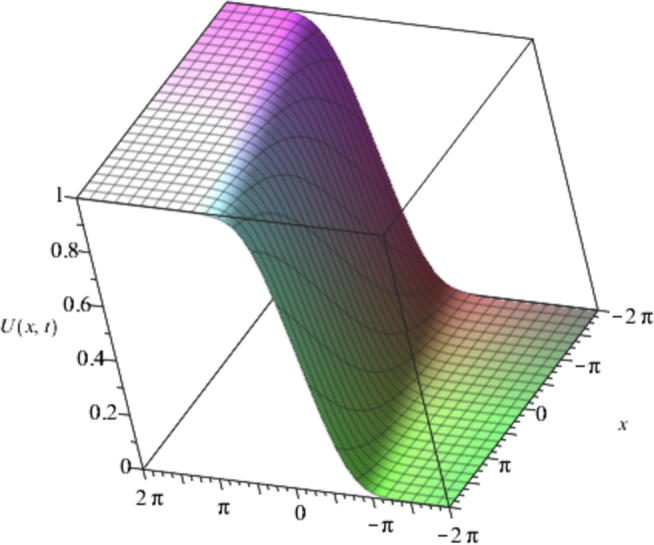
Solution plots of the multivariate series (Solitary solution) obtained in eq. (61) using the proposed EPDTM with ***α* = −1**, ***β* = 2**.

**Figure 3:**
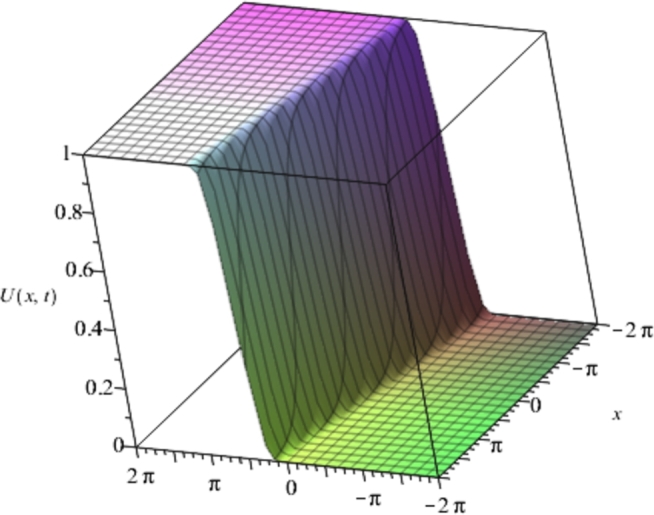
Solution plots of the exact solution/closed form solution in eq. (62) with ***α* = 3**, ***β* = 6**.

**Figure 4:**
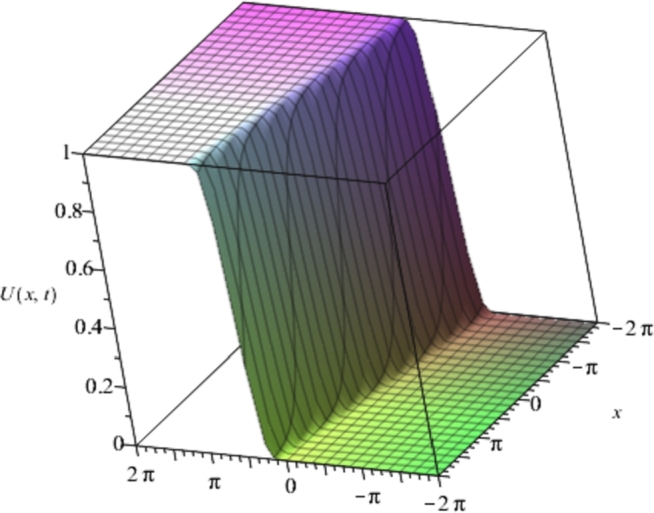
Solution plots of the multivariate series (Solitary solution) obtained in eq. (61) using the proposed EPDTM with ***α* = 3**, ***β* = 6**.

**Figure 5:**
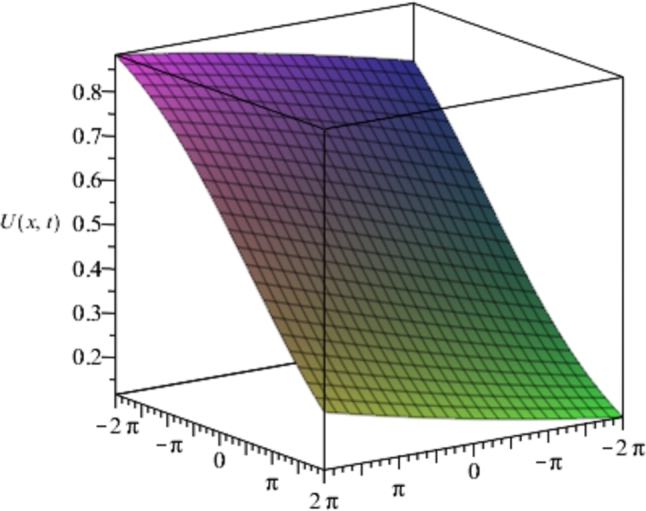
Solution plots of the exact solution/closed form solution in eq. (62) with ***α* = 0.5**, ***β* = 0.01**.

**Figure 6:**
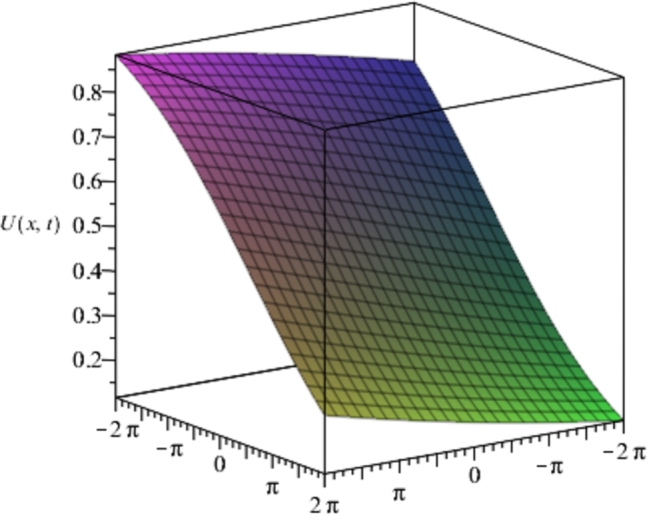
Solution plots of the multivariate series (Solitary solution) obtained in eq. (61) using the proposed EPDTM with ***α* = 0.5**, ***β* = 0.01**.

**Figure 7:**
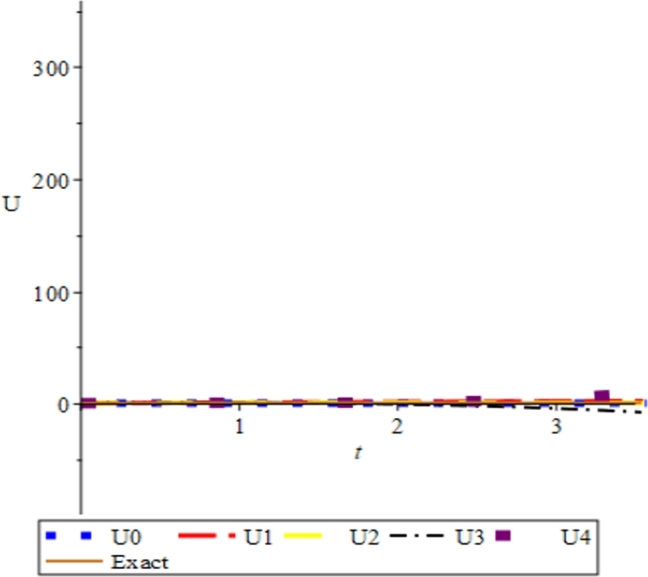
Convergence plots of the multivariate series (Solitary solution) obtained in eq. (61) using the proposed Elzaki projected differential transform (EPDTM) with ***x* = 1**, ***α* = −1**, and ***β* = 2**.

**Figure 8:**
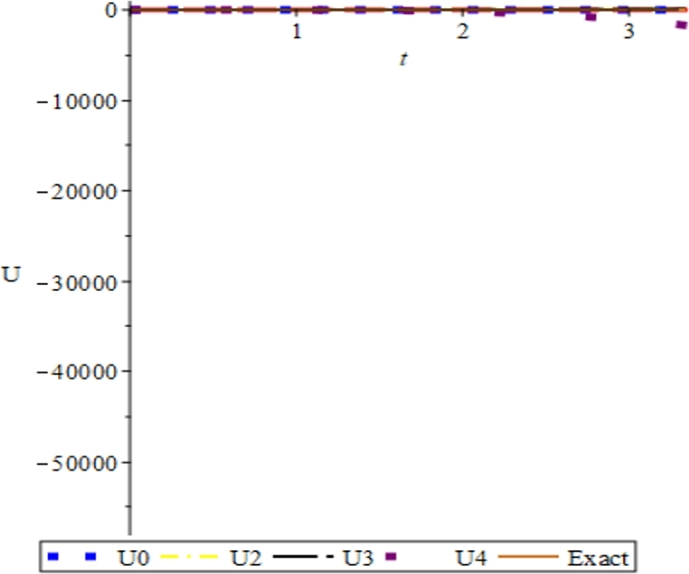
Convergence plots of the multivariate series (Solitary solution) obtained in eq. (61) using the proposed Elzaki projected differential transform (EPDTM) with ***x* = 1**, ***α* = 3**, and ***β* = 6**.

**Figure 9:**
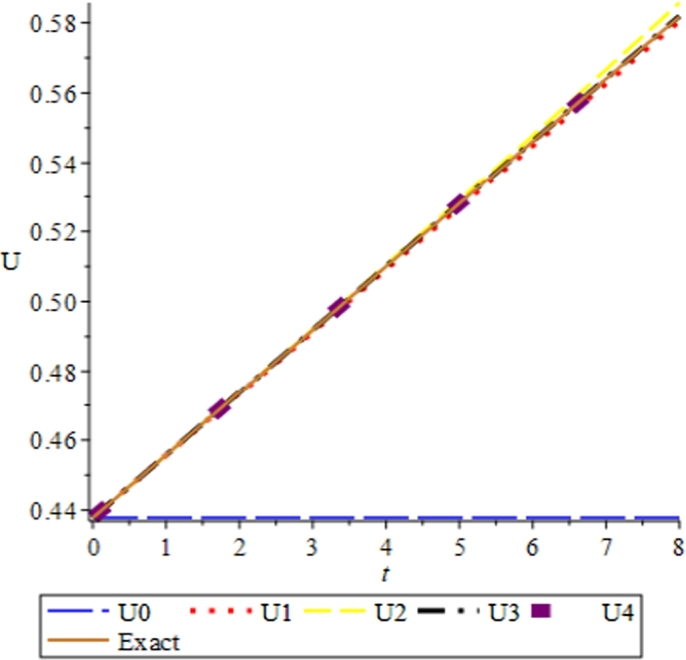
Convergence plots of the multivariate series (Solitary solution) obtained in eq. (61) using the proposed Elzaki projected differential transform (EPDTM) with ***x* = 1**, ***α* = 0.5**, and ***β* = 0.01**.

**Figure 10:**
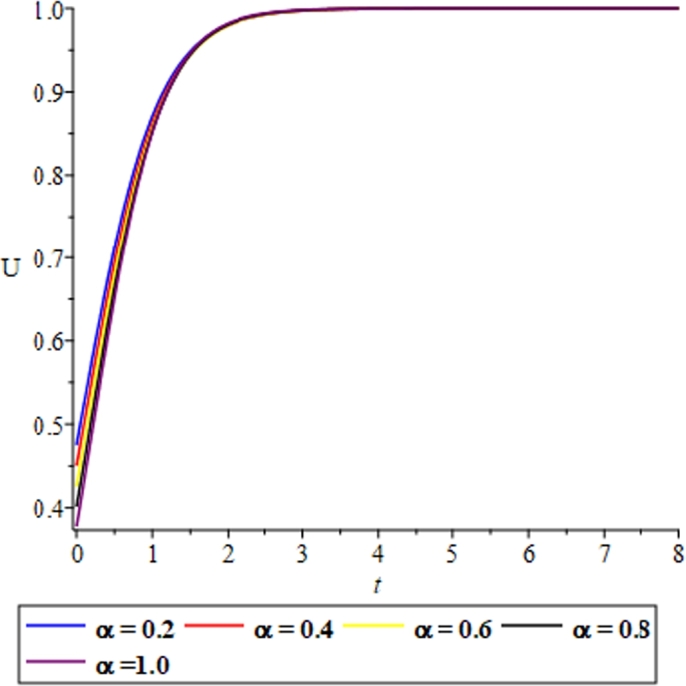
The effect of the reaction coefficient *α* and the fluid-like behaviour of the solitary solution in equation (61) showing the validity of the solution.

**Figure 11:**
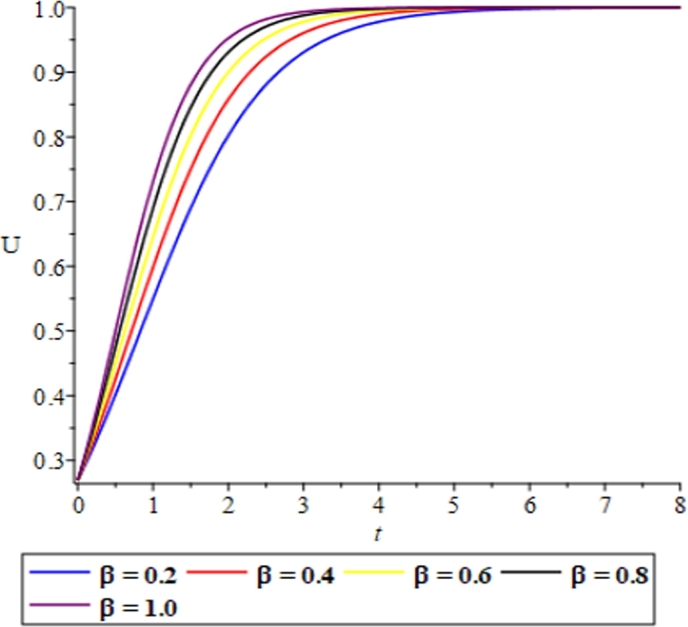
The effect of the diffusion coefficient *β* and the fluid-like behaviour of the solitary solution in equation (61) showing the validity of the solution.

## 9 Discussion of findings

In this research work, we have proposed and implemented an unprecedented hybrid scheme viz: Elzaki integral transform coupled with the projected differential transform which is a modified or improved differential transform method in obtaining a solitary solution to the generalized Burgers-Fisher's equation which is prominent and pertinent in financial mathematics, reactive mechanisms, fluid mechanics, turbulence theory, and applied sciences generally.

Elzaki integral transform is a modification of the Sumudu and Laplace transform, an exact method for linear partial differential equations, which facilitates the process of solving differential equations, and has also solved equations that Sumudu and Laplace failed to solve. The projected differential transform (PDTM) on the other hand is a modification of the well known differential transform (DTM), an asymptotic method which decomposes highly nonlinear terms in a simplified series form without the need of an embedding parameter like that of homotopy perturbation method (HPM), expansion of special polynomials like that of Adomian decomposition method (ADM) and HPM, comparison of powers or coefficients of special polynomials like the HPM and ADM, with less computational stress, yet yielding highly convergent and accurate results.

The novelty of this research thus lies in merging these two improved and powerful methods to provide a solitary solution to a highly nonlinear partial differential equation viz: the Burgers-Fisher's equation.

The solitary solution was obtained in form of an infinite multivariate Taylor polynomial which rapidly converges to the exact solution of the problem. The exact solution here is that obtained using prominent analytical techniques like the first integral method and the Adomian decomposition method [51], [65], [66].

In order to certify the validity of our method with the normal analytical methods in the literature, a table of comparison showing the exact results, Elzaki projected differential transform results (EPDTM) and the absolute error has been presented for α=−1,β=2, α=3,β=6, and α=0.5,β=0.01; of which shows a flawless convergence.

Furthermore, the solution plots in three dimension (3D) and convergence plots of the respective solutions obtained using EPDTM show that the proposed method agreed excellently with the exact solution of the problem.

Figure 1, Figure 2, Figure 3, Figure 4 show a kink solution plot (kink waves) that rises or descends from one asymptotic state to another and also approaches a constant at infinity. While Figure 5, Figure 6 show a compacton solution as a result of the finite tail and wavelength, with a remarkable soliton property that causes it to re-emerge itself back to the coherent shape after colliding with other compactons.

In the quest of certifying the validity of the proposed EPDTM in providing a solitary solution to the Burgers-Fisher's equation, graphical illustrations showing the variation effects of the reaction and diffusion coefficients respectively for the equation (61) have been presented; and this shows the fluid-like behaviour of the model. (Figure 7, Figure 8, Figure 9, Figure 10, Figure 11.)

## 10 Conclusion

Sequel to the results obtained using our proposed Elzaki projected differential transform method (EPDTM); the simplicity and less computational work of the method; the convergence of these results when compared with existing analytical methods in the literature via tables, and convergence plots. We can hence conclude that the Elzaki projected differential transform method is valid, reliable and highly efficient in solving and conveying analyses on reaction-diffusion equations and wider classes of PDEs.

Furthermore, due to the reliability and efficiency of this method, we hereby recommend this proposed method (EPDTM) in providing exact solutions, generalized solutions, and also solitary wave solution on equations in Fluid mechanics, reaction-diffusion theory, turbulence theory, nonlinear dynamics and also for parabolic PDEs like that of the Burgers-Fisher's.

## Declarations

### Author contribution statement

Akinfe Timilehin K.: Conceived and designed the experiments; Performed the experiments; Analyzed and interpreted the data; Contributed reagents, materials, analysis tools or data; Wrote the paper. Loyinmi Adedapo C.: Analyzed and interpreted the data; Contributed reagents, materials, analysis tools or data.

### Funding statement

This research did not receive any specific grant from funding agencies in the public, commercial, or not-for-profit sectors.

### Data availability statement

No data was used for the research described in the article.

### Declaration of interests statement

The authors declare no conflict of interest.

### Additional information

Supplementary content related to this article has been published online at https://doi.org/10.1016/j.heliyon.2021.e07001.

No additional information is available for this paper.

## Supplementary material

The following Supplementary material is associated with this article:

HELIYON-D-20-04652R2_appendix.docx Elzaki Table of Transform for some functions. HELIYON-D-20-04652R2_appendix.docx
