# Supplementary material for: A solitary wave solution to the generalized Burgers-Fisher's equation using an improved differential transform method: A hybrid scheme approach
Source: Heliyon. 2021 May 12;7(5):e07001. doi: 10.1016/j.heliyon.2021.e07001 (PMC8180614; doi:10.1016/j.heliyon.2021.e07001)
Supplement: HELIYON-D-20-04652R2_appendix.docx — Elzaki Table of Transform for some functions. [file mmc1.docx]

**Appendix**

Elzaki Table of Transform for some functions

| $f(t)$ | $E\left[f(t)\right]=T(v)$ |
| --- | --- |
| $1$ | $v^{2}$ |
| $t$ | $v^{3}$ |
| $t^{n}$ | $n!v^{n+2}$ |
| $e^{at}$ | $\frac{v^{2}}{1-av}$ |
| $te^{at}$ | $\frac{v^{3}}{\left(1-av\right)^{2}}$ |
| $\sin at$ | $\frac{av^{3}}{1+a^{2}v^{2}}$ |
| $\cos at$ | $\frac{u^{2}}{1+a^{2}v^{2}}$ |
| $\sinh at$ | $\frac{av^{3}}{1-a^{2}v^{2}}$ |
| $\cosh at$ | $\frac{av^{2}}{1-a^{2}v^{2}}$ |
| $e^{at}\sin bt$ | $\frac{bv^{3}}{\left(1-av\right)^{2}+b^{2}v^{2}}$ |
| $e^{at}\cos bt$ | $\frac{\left(1-av\right)v^{2}}{\left(1-av\right)^{2}+b^{2}v^{2}}$ |
| $t\sin at$ | $\frac{2av^{4}}{1+a^{2}v^{2}}$ |
| $\frac{t^{a-1}}{\Gamma\left(a\right)},a=0$ | $v^{a+1}$ |
| $\frac{t^{n-1}e^{at}}{\left(n-1\right)!},$ $n=1,2,...$  | $\frac{v^{n+1}}{\left(1-av\right)^{n}}$ |
| $J_{0}\left(at\right)$ | $\frac{v^{2}}{\sqrt{1+av^{2}}}$ |
| $H\left(t-a\right)$ | $v^{2}e^{-\frac{a}{v}}$ |
| $\delta\left(t-a\right)$ | $ve^{-\frac{a}{v}}$ |
